# Attitudes surrounding the disclosure of mental illness

**DOI:** 10.1192/bjo.2021.767

**Published:** 2021-06-18

**Authors:** Brishti Sengupta, Pritha Dasgupta

**Affiliations:** 1 Dollar Academy; 2 Stratheden Hospital, NHS Fife

## Abstract

**Aims:**

To survey the effect of COVID-19 on mental health of both medical professionals and the general population, as well as attitudes surrounding the disclosure of mental illness.

**Method:**

An online survey comprised of two questionnaires, one for medical professionals and one for the general population, were conducted via social media. Both questionnaires asked respondents of the effect of COVID-19 on their mental health, and the former asked respondents about the effect of COVID-19 on their patient group's mental health. The questionnaires went on to ask respondents about their attitudes to mental health disclosure in various scenarios, to varying groups of people. The general population group was also asked how they would react if someone else disclosed their mental illness to them.

**Result:**

The questionnaire for the medical professionals gained 62 respondents and the one for the general population had 122 respondents, with responses from multiple nations. Overall, COVID-19 has affected everyone's mental health to a degree, and all groups had reservations about disclosing their mental health issues to others. The medical professionals were especially reluctant to disclose mental illness to their patients, but were more comfortable when it came to disclosing mental illness to colleagues. The general population, however, was much more reluctant to disclose mental health issues to their colleagues. The general population were, on the whole, willing to listen to and help anyone who came to them with mental health concerns. Both groups surveyed showed reluctance toward disclosure to the wider community.

**Conclusion:**

COVID-19 appears to significantly affect not only physical health, but mental health as well. There is at least some degree of stigma surrounding the disclosure of mental health issues. While most would be happy to help anyone who came to them with their mental health problems, there seems to be an attitude shift when people must contend with mental health issues of their own.

